# Regenerative Therapies for Basal Thumb Arthritis—A Systematic Review

**DOI:** 10.3390/ijms241914909

**Published:** 2023-10-05

**Authors:** Sophie Hasiba-Pappas, Lars-P. Kamolz, Hanna Luze, Sebastian P. Nischwitz, David B. Lumenta, Raimund Winter

**Affiliations:** 1 Research Unit for Tissue Regeneration, Repair and Reconstruction, Division of Plastic, Aesthetic and Reconstructive Surgery, Department of Surgery, Medical University of Graz, Auenbruggerplatz 5, A-8036 Graz, Austria; sophie.hasiba@gmail.com (S.H.-P.); lars.kamolz@medunigraz.at (L.-P.K.); david.lumenta@medunigraz.at (D.B.L.); 2 COREMED—Cooperative Centre for Regenerative Medicine, Joanneum Research GmbH, Neue Stiftingtalstr. 2, A-8010 Graz, Austria

**Keywords:** carpometacarpal arthritis, rhizarthrosis, regenerative therapy, platelet-rich plasma, fat grafting, lipofilling, phototherapy, LLLT, chondrocyte transplant

## Abstract

Basal thumb arthritis is a painful and debilitating pathology that can severely reduce a patients’ quality of life. Common therapies include oral pain control, local steroid injections and/or surgery. Yet, therapeutic data on long-term improvement and even cartilage repair are scarce. This review aims to present the currently available literature on novel therapies for basal thumb arthritis, including platelet-rich plasma (PRP), fat grafting and phototherapy, and investigate their potential efficacy. The entire OVID database and PubMed were searched for studies containing the topics PRP injection, lipofilling, laser treatment and regenerative treatment for carpometacarpal arthritis. Seven studies on the effect of fat tissue on basal thumb arthritis were found. Four authors reported on PRP injections, one RCT examined a combinational treatment of PRP and fat grafting, another phototherapy for the thumb joint and one prospective trial on chondrocyte transplantation was found. Pain improvement and decreased impairment were reported in the majority of PRP and/or fat grafting studies as well as after chondrocyte implantation. Phototherapy did not significantly improve the condition. This review revealed that only limited data on regenerative therapies for carpometacarpal arthritis are currently available, yet PRP and lipofilling show promising results and merit further investigation.

## 1. Introduction

Arthritis of the basal thumb joint, also known as carpometacarpal arthritis (CMC-1 arthritis), is a highly debilitating disease with a high global prevalence [1,2,3]. The patient demographic predominately consists of peri- or postmenopausal women [1], however, men and women of all ages may be affected [2]. 

Osteoarthritis (OA) causes synovial inflammation, progressive cartilage destruction and changes in the subchondral bone structure, which ultimately lead to chronic pain [4]. The thumb is crucial for grasping and strength exertion of the entire hand. Therefore, chronic pain and cartilage degradation result in significant impairment of the entire hand function. The permanent discomfort, loss of function and constant need for pain medication substantially reduce the patients’ quality of life [5]. Thus, finding effective treatment options is of high priority in OA research.

Common therapies for CMC-1 arthritis mainly include conservative management with steroids or non-steroidal anti-inflammatory drugs (NSAIDs) [3] and surgical treatment, such as ligament reconstruction, tendon interposition, resection arthroplasty, joint replacement or arthrodesis [6]. Steroids and NSAIDs, on the one hand, can provide temporary pain relief and inhibit further inflammation but are associated with significant side effects when administered over a long period of time [7]. Surgical treatment, on the other hand, may provide long-term pain relief and improve function, yet it carries the risk of developing postoperative complications like joint stiffness, dystrophia, nerve damage or the need for surgical revision [6]. 

While both anti-inflammatory medication and surgical interventions provide symptomatic relief and may delay joint destruction, neither can promote cartilage repair. Thus, the development of regenerative therapies and tissue-based techniques has been at the center of research on cartilage repair [8].

Platelet-based therapies have emerged as a popular treatment option for osteoarthritis. Reportedly, platelet-rich plasma (PRP) influences inflammatory cytokines, enzymes and transcription factors that play crucial roles in cartilage repair. Recent studies have shown positive effects of PRP in both preclinical and clinical settings [6].

Applications of adipose-tissue-derived stem cells (ADSCs) were investigated in different types of OA. ADSCs are mesenchymal stem cells that can be found in adipose tissue and have the potential to differentiate into various tissues, including chondroblasts and chondrocytes [9]. Both chondroprotective and anti-inflammatory effects of ADSCs were described in the literature [10]. Extraction and isolation of these stem cells from lipoaspirates are complex processes and an extensive ethical review is required before research in regulated environments can be conducted [11]. Direct fat grafting (FG) after tissue harvest without additional extraction of adipose cells, however, can be safe, easy and cost-efficient [12]. High numbers of ADSCs with chondroprotective potential were observed in lipoaspirates [13], spawning the theory that fat grafting itself may have regenerative effects on OA [14]. 

Low-level laser therapy (LLLT) is less explored than PRP or adipose cells. Yet, a few studies reporting on increased chondrocyte differentiation [15] or anti-inflammatory effects [16] in experimental models have sparked interest in LLLT as another treatment option for OA.

While multiple studies and reviews discussing the potential effect of these therapies on osteoarthritis of the knee exist [17,18], the literature on CMC-1 arthritis is quite scarce and only a few systematic reviews investigated the possible impact on arthritis of the basal thumb joint. 

The aim of this study was to (1) review the currently available literature on therapies with regenerative potential for CMC-1 arthritis, (2) provide an update on PRP and fat grafting for basal thumb arthritis and (3) present and discuss their outcomes.

## 2. Material and Methods

### 2.1. Literature Search

We searched the databases PubMed and OVID (all OVID databases including Medline, Embase and the Cochrane Library) for studies on regenerative therapy methods, including, but not limited to, platelet-rich plasma, fat grafting and phototherapy, for the treatment of carpometacarpal arthritis. A systematic literature search in line with PRISMA guidelines was performed until the 15th of August in 2023. The protocol for this systematic review was registered on INPLASY (registration number INPLASY202380131).

All search terms applied for the inquiry are displayed in Table 1.

### 2.2. Inclusion and Exclusion Criteria 

The search focused on studies on novel and/or regenerative therapeutic approaches for the treatment of carpometacarpal arthritis. Only studies that reported on the treatment of the basal thumb joint were included in this review. Case reports and review articles were excluded after the search. Since the aim of this research was to explore the current clinical state of knowledge, only human clinical studies were included and in vitro investigations were excluded.

### 2.3. Data Extraction

**Figure 1:**
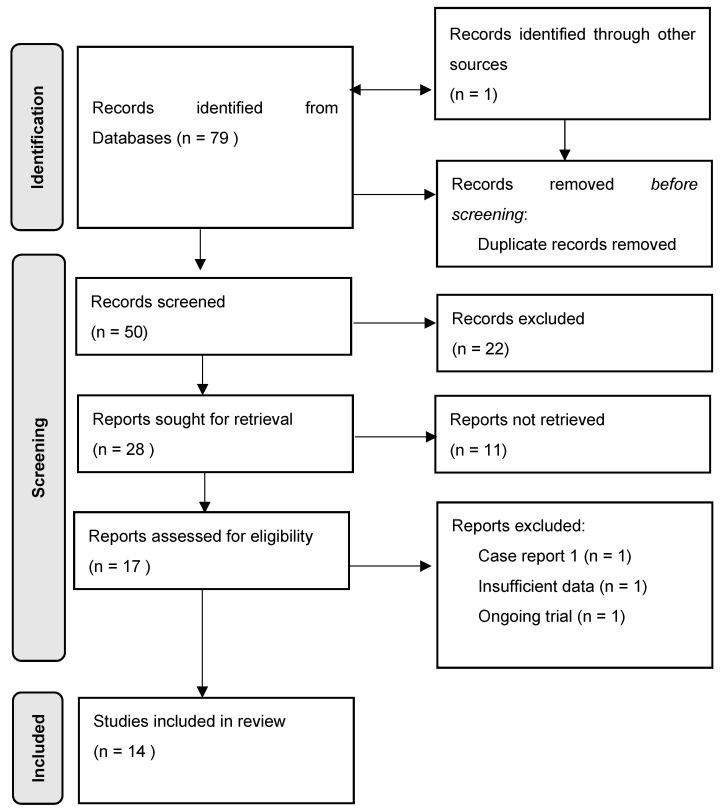
Flow diagram describing the study selection process.

Following the assembly of all findings from the online search, duplicates were manually removed. All remaining titles and abstracts were screened for eligibility, followed by a full text review. The detailed selection process is further displayed in the flow diagram, Figure 1.

## 3. Results

A total of 79 studies were obtained through the research process. One study was found through additional sources. Fifty publications remained after deduplication. Fourteen studies were deemed eligible and therefore included in this systematic review.

The search yielded seven publications on fat grafting for basal thumb arthritis [14,19,20,21,22,23,24], three of which were prospective studies [14,21,24]. Four groups of authors reported on platelet-based treatment of basal thumb arthritis with platelet-rich plasma injections [25,26,27,28]. One randomized controlled trial (RCT) investigated fat grafting, platelet-rich plasma and a combination of the two methods [29]. Low-level laser therapy of the finger joints, including the basal thumb joint, was explored in one RCT [30]. One group of authors conducted a prospective study on autologous chondrocyte transplantation in ten cases of carpometacarpal arthritis [31].

The patient collective was predominantly female; especially women in the peri- or postmenopausal phase. The mean age among all included studies varied between 52 and 65 years. The severity of the osteoarthritis (OA) of the thumb basal joint was graded according to the Eaton–Littler classification. Some of the studies treated patients of all OA grades (I–IV), whereas others chose not to include cases of severe OA. The mean follow-up time showed a high variation, depending on the study type. The shortest time period until the last postoperative examination was three months, while the longest mean follow-up time of eight years was reported in a prospective study [31]. Five out of twelve studies that investigated fat and/or PRP injections as treatment options for CMC-1 arthritis had control groups [19,21,28,29,30]. In most cases, cortisone, hyaluronic acid (HA) or saline were applied in the comparative collective. One group of authors compared intraarticular lipofilling to surgical resection arthroplasty [19]. In the case of low-level laser treatment, sham or “placebo” LLLT was applied to the patients belonging to the comparative collective [30]. The study characteristics are displayed in Table 2. 

### 3.1. Outcome

#### 3.1.1. Fat Grafting 

In total, eight studies investigated the effect of fat grafting for trapeziometacarpal arthritis [14,19,20,21,22,23,24,29]. Fat was harvested from a suitable donor site (thigh, lower abdomen) and prepared for injection. Some authors opted for centrifugation according to the Coleman technique, others preferred mechanical homogenization. In one case, the fat was harvested and stored for ten minutes to achieve sedimentation [29]. After preparation, between 1 and 2 mL (depending on the study and on the individually varying space in the thumb joint) was injected directly into the joint.

The most frequently used parameters for outcome assessment were pain (measured by the visual analogue scale, VAS, or numeric rating scale, NRS), functional impairment (measured by the Disabilities of the Arm, Shoulder and Hand Questionnaire, DASH) and pinch and grip strength. Patient satisfaction was reported by four authors [19,20,22,23]. 

While all patients treated with lipofilling reported a decrease in pain in terms of lower VAS or NRS scores, not all results were statistically significant. Statistical significance was often associated with the OA stage according to Eaton–Littler or whether the pain level in the joint was measured at rest or in motion. Haas et al. [22], for instance, reported a statistically significant VAS decrease from 6.6 to 3.7 under stress, whereas pain improvement at rest did not show significance at the last follow-up exam. Herold et al. [14] reported similar results from a prospective study published in 2017. Significant pain improvement was achieved for joint movement and/or when pressure was applied to the CMC-1 joint. Moreover, the authors observed that patients who suffered from stage II or III CMC-1 arthritis benefited more from the intervention than those with severe rhizarthrosis, demonstrated in the VAS scores from stage IV participants, which were not significantly better after lipofilling. Winter et al. [29] also investigated the disparities in pain decrease according to different OA stages and found that grade III participants benefited significantly more from the treatment than stage II patients. Similarly to Herold et al. [14], severe cases profited the least. In a previous study conducted by Haas et al. [21], patients who suffered from grade IV CMC-1 arthritis were eliminated in the selection process. In this prospective pilot study, 24 patients with basal thumb arthritis with stages I–III were injected with either autologous fat or cortisone. Participants from the fat group reported a statistically significant pain reduction both at rest and under stress, which held up until the last postoperative examination. Froschauer et al. [20] and Meyer-Marcotty et al. [24] did not conduct a separate analysis for pain levels at rest or under stress. Both groups of authors observed significant pain relief in all treated patients. Meyer-Marcotty et al. [24] even stated that the mid-term results of their prospective trial were highly significant in terms of pain reduction as well as improvement of the hand function, measured by the DASH score.

The DASH or Q-DASH, which is the shortened version of the DASH questionnaire, was used as an outcome measure in seven out of eight studies on fat grafting. Most authors reported a significantly lower (Q-)DASH score, indicating a lower degree of impairment due to the condition after lipofilling. In accordance with the VAS results, Herold et al. [14] observed a superior effect of the fat transfer for mild to moderate OA, based on the subgroup analysis, which showed that only stage II patients presented with significantly improved DASH scores at all postoperative examination timepoints. 

Subjective and/or objective pinch and grip strength were unaffected or not significantly improved in most studies which reported on this outcome measure. Two groups of authors did not state the preoperative pinch and grip scores, however, they wrote about no relevant improvement when compared to either the comparative group or the preoperative score [28,30]. Some patients reported an initial decline of grip and pinch strength directly after the intervention; however, this was only temporary and grip and pinch strength slowly recovered over the follow-up period. Herold et al. [14] were the only authors to observe significantly improved pinch strength in stage II patients 12 months postoperatively. 

Patient satisfaction, if reported, was assessed with different outcomes depending on the study. Erne et al. [19] asked participants to rate their contentment with the procedure in points, with 1 indicating low and 10 indicating high satisfaction. Overall, patients rated the procedure with eight points. Froschauer et al. [20] and Haas et al. [22] calculated the satisfaction rate in percentages. The former observed a 68% satisfaction rate, whereas the latter stated that 73% of all participants would reportedly undergo the procedure again and 84 % would recommend it to a friend. In the pilot study conducted by Herold et al. [23] in 2014, all patients were content with the treatment. 

#### 3.1.2. PRP

The effect of platelet-rich plasma injections into the CMC-1 joint was investigated in five studies [25,26,27,28,29], two of which were RCTs [26,29]. To prepare the PRP, venous blood was drawn from the patients and then centrifuged according to the instructions of the systems used. Two groups of authors reported double centrifugation [26,28]. The final product was then injected directly into the joint. Winter et al. [29] chose to add calcium chloride as a PRP activator prior to injection, whereas Malahias et al. [26] reportedly injected non-activated PRP. Other authors provided no information on the form of preparation. In three out of five publications, two treatment sessions took place in a 2-to-4-week interval [25,26,27]. Two groups of authors treated patients with one single PRP injection [28,29]. After the procedure, participants in the RCT by Winter et al. [29] were instructed to wear only a non-immobilizing dressing for a few days. 

Sabah et al. [28] evaluated the potential effect of PRP by assessing pain, pinch and grip strength as well as the Australian and Canadian Hand Index (AUSCAN), which is used to evaluate the hand function by applying three subscores (pain, stiffness and function) clinically. The authors reported significant improvement for all outcome assessment scales at the 4-week follow-up for all study groups (PRP, HA, cortisone). The observed statistical significance of both PRP and cortisone did not extend beyond the 12-week follow-up. Swärd et al. [27], who investigated the effect of PRP injections on twenty-one CMC-1 joints and eight scaphotrapeziotrapezoid (STT) joints, could not detect any statistically significant improvement for pain (NRS), function (patient-rated wrist evaluation score, PRWHE) or grip as well as pinch strength at any follow-up. By contrast, Malahias et al. [26] and Loibl et al. [25] noticed statistically significant pain improvement six months after the second PRP injection. Statistically significant findings were not detected for the DASH score or pinch and grip strength. The therapeutic effects on the Mayo wrist score were again significantly better six months postoperatively, indicating a functional benefit. In addition to the pain relief, the patients in the RCT by Malahias et al. [26] also observed significantly lower DASH scores. Moreover, the group treated with PRP showed a significantly higher satisfaction rate than the one with cortisone injections. 

The PRP group in the RCT by Winter et al. [29] showed no statistically significant improvement when compared to the other study groups.

#### 3.1.3. PRP and Fat Grafting 

Only one study investigated the effects of a combinational therapy of basal thumb arthritis with platelet-rich plasma and lipofilling in a randomized controlled setting. Winter et al. [29] injected 25 patients with a mixture of 0.75 mL autologous fat and 0.75 mL centrifuged activated PRP. The results from the FG + PRP group were compared to three equally sized groups, whose participants either received fat, PRP or saline. Patients from all four groups were advised to wear a bandage for a few days. Both pain at rest and under motion were significantly reduced after the intervention. The pain assessment (NRS) and disability score (Q-DASH) in the FG + PRP group were better than the ones in the other groups. Additional analysis revealed that patients with grade III OA benefited the most from the treatment in all intervention groups. Pinch and grip strength were not affected in a statistically significant manner [29]. 

#### 3.1.4. Low-Level Laser Treatment

We extracted just one publication on the effects of phototherapy: Brosseau et al. [30] applied low-level laser treatment in patients suffering from OA of the finger joints, including CMC-1 arthritis, in a double-blinded RCT. The control group received sham LLLT. An Eriel laser with gallium aluminum arsenide (GaAIAs) as the active medium was used for the active LLLT cohort. Treatment with a chosen modulation of 20 Hz was applied via a probe that was positioned at 90 degrees to the joint and had direct skin contact. Participants had three treatment sessions per week over the course of six weeks total. Each session lasted twenty minutes. Maintenance of the device was conducted regularly by a qualified technician [30]. 

Pain was assessed via VAS and AUSCAN scores, none of which showed statistically significant differences between the study and control groups during follow-up. The range of motion (ROM), joint flexion and morning stiffness showed no statistically significant improvement in the active LLLT group when compared to the sham LLLT cohort. However, thumb opposition was significantly improved in the active LLLT group (significant reduction in distance between thumb and fifth metacarpal). No DASH scores were obtained in this study [30].

#### 3.1.5. Autologous Chondrocyte Transplantation

Messina et al. [31] conducted a prospective study on ten cases of stage II or III basal thumb arthritis. Patients received a combined treatment of CMC arthroplasty and simultaneous implantation of autologous chondrocytes. First, 3–4 mm cartilage fragments were harvested from either the wrist or the elbow (arthroscopically or by an open technique) prior to the transplantation. The harvested cartilage cells were grown on a collagenous biphasic matrix in a laboratory. After 3 weeks, a chondrocyte-augmented scaffold was ready for transplantation to the affected joints. Surgical treatment varied individually among patients. While all participants had a synovectomy and joint debridement, partial trapeziectomy and ligament reconstruction were only performed in severe cases. A long-term follow-up of up to eleven years was possible for most participants [31]. 

Outcome was measured with VAS, DASH, Mayo and PRWE scores. Both pain and functional impairment were significantly decreased postoperatively. Grip and pinch strength were increased in both groups, but only the pinch strength values were significantly better compared to the preoperative scores. In nine out of ten cases, results were regarded as “excellent” according to the Mayo wrist score, while one case was described as a “good” result. Postoperative PRWE score was 7.7, while preoperative data were not presented. All participants regained a full ROM after the procedure [31]. 

Postoperative MRI and CT scans were performed to evaluate the intervention (i.e., the quality of the novel cartilage), however, no distinction between soft tissue and cartilage was possible with the performed imaging studies [31].

### 3.2. Complications 

Overall, no major complications were observed in any of the reports. 

Winter et al. [29] reported three serious adverse events—none of them associated with the study treatments—and only study-related complication, which was a hematoma at the injection site from one of the study treatments (not explicitly stated). Similarly, one minor hematoma was found in a fat grafting patient in the study by Haas et al. [22] after liposuction. Another minor complication after autologous fat transfer was observed by Herold et al. [14]. Two patients complained of paresthesias around the supply area of the superficial radial nerve branches with the symptoms being temporary and resolving without further intervention. Loibl et al. [25] reported one minor event after PRP injection. One patient suffered from a palmar ganglion, which receded shortly thereafter without additional treatment. Overall, both PRP and FG were described as safe treatment options. 

Three patients dropped out of the LLLT trial due to undisclosed adverse events and were therefore not included in the final calculations. Two cases were reported to OHREB by Brousseau et al. [30]. One menopausal participant stated that her periods seem to reappear during the course of the low-level laser treatment, and one patient suffered from an erythema on the forearm and wrist after the first session. No other complications or side effects were observed throughout this trial [30]. 

All outcome measures and comlications are portrayed in Table 3. 

## 4. Discussion

The aim of this systematic review was to present the current clinical research on regenerative therapies for carpometacarpal arthritis. The majority of the trials focused on the outcome of fat grafting for CMC-1 arthritis. 

Lipofilling significantly reduced the disability in most studies. The impact on pain relief was more noticeable in severe OA cases (stage II or III). The mechanism of action of autologous fat tissue has not been fully understood. Fat may serve as a buffer in the joint space, thereby providing pain relief. In addition, autologous fat may have regenerative properties due to the high number of ADSCs found in the lipoaspirates [14]. Standard lipofilling is an alternative to ADSC isolation, since it is an easy, low-cost, safe procedure and unlike stem cell isolation has no regulatory restrictions imposed. However, no literature evidence supports that ADSCs contained in the lipoaspirate can achieve the same effects in a clinical setting as isolated ADSCs did in vitro. One concern is the inconsistency of fat graft survival, which is crucial for ADSC differentiation. Another is the durability of fat grafting effects on pain. A systematic review on PRP or FG was conducted by the authors involved in this work in 2021 [2], and the lack of long-term results was one of the main concerns back then. Two studies on CMC-1 lipofilling were published after the 2021 review: Meyer-Marcotty et al. [24] in a mean follow-up of 45 months observed significant improvement in these patients. Winter et al. [29] reported the statistically significant outcome of a combination therapy of PRP and fat grafting vs. two other modalities over a mean follow-up of 24 months. This synergistic effect seems to result from PRP enhancing graft survival and secretion of growth factors like vascular endothelial growth factor (VEGF) and modulating immunogenic responses, pointing to the regenerative benefits of a combined treatment [12,32,33].

The effects of PRP alone were contradictory. While Malahias et al. [26] and Loibl et al. [25] reported statistically significant improvement combined with high patient satisfaction, other authors did not. However, comparability of the included studies was diminished by the incomplete datasets. Despite the number of published reports on PRP injections for rhizarthrosis treatment almost doubling over the past years, substantial RCTs with long-term follow-up have not become available at the conclusion of this review. 

The study with the longest follow-up was conducted by Messina et al. [31]. The authors provided the only research on autologous chondrocyte transplantation for CMC-1 arthritis. Eight years after the procedure, significant improvements in pain and function were still observed. However, unlike the other studies, Messina et al. [31] combined the treatment with surgical interventions like partial trapezium resections or ligament reconstructions, rendering comparability to less invasive treatments alone or the interpretation of the impact of the implanted cartilage scaffold itself difficult. Data on chondrocyte transplantation for arthritis to other joints is also limited and more research on this topic is needed to obtain long-term results on cartilage graft survival [34]. As tissue engineering is gaining relevance in both cartilage and bone repair, various fields are being investigated in terms of their regenerative potential. In addition to cell-based methods like chondrocyte transplantation, growth factors, mechanical stimuli and biomaterial scaffolds [35] have been explored in in vitro settings [36]. 

Low-level laser treatment did not produce any statistically relevant results except for the increased grip strength after six weeks of treatment [30]. However, as this was the only study examining phototherapy on basal thumb arthritis it is difficult to make recommendations in this literature review. So far, LLLT has mainly been applied in cases of knee OA. Motivated by conflicting opinions surrounding the efficacy of LLLT for knee OA, Stausholm et al. [17] conducted a systematic review and meta-analysis including 22 randomized controlled trials. The authors concluded that phototherapy does reduce pain and disability in OA of the knee. According to this research, the dose of the laser (i.e., wavelength and pulsation) highly influences the therapeutic success. Therefore, it may be worthwhile to conduct further studies on LLLT for CMC-1 arthritis to generate more data and to identify the appropriate laser dose for the thumb joint. In fact, Medina-Porqueres et al. [37] recently registered an RCT on laser treatment for stage I and II rhizarthrosis and published the study protocol online. Another study on this topic was registered to the Cochrane Register of Controlled Trials in 2021, and both projects may provide further information and build a foundation for future research. 

Although trends towards a beneficial outcome were observed for PRP injections and lipofilling in this systematic review, the currently available literature is too scarce to provide adequate scientific evidence for either of these therapies at this stage. We believe this research will provide future beneficial regenerative therapies of PRP and autologous fat grafting and support their exploration in more randomized controlled trials. 

### Limitations 

One limitation was the heterogeneity among trials. Especially, the preparation process of PRP showed high variability in the centrifugation (once or twice), activation (activated versus non-activated PRP) and number of treatments. Fewer disparities were observed in treatment protocols on fat grafting, yet the preparation process for the injected fat varied as well.

Another limitation was the scarcity of trials found on this topic. Although the number of studies on PRP or fat grafting may suffice to compare between different study protocols and observe trends, only one publication on LLLT was found. Therefore, one cannot make a general statement on the efficacy of phototherapy for CMC-1 arthritis. Similarly, the sole well-conducted prospective study on chondrocyte transplantation does not allow us to draw conclusions on potential benefits in the treatment of basal thumb arthritis in the context of this literature review. 

## 5. Conclusions

Evidence-based literature on PRP, lipofilling and LLLT for rhizarthrosis in a clinical setting is scarce. Both fat grafting and platelet-rich plasma injections improved pain and reduced functional impairment in the included studies, mostly indicating trends and less so statistical significance. The combination of PRP and fat grafting may be of interest for future trials and to obtain synergistic results from cell-based as well as clinically focused articles. Only one study on phototherapy for CMC-1 arthritis did not show relevant changes in pain or strength. Ongoing trials may produce different results, based on the literature on LLLT on knee OA. One single study on autologous chondrocyte transplantation yielded excellent results with no comparable literature available. 

The therapies described in this study could provide symptomatic relief and may have regenerative potential in OA treatment, however, this must be further investigated in larger RCTs. 

## Figures and Tables

**Table 1:** Search strategy.

(1) ^1^		(2)	(3)	(4)	(5)
	***AND***				
*Carpometacarpal arthritis*			LLLT		
***OR***			*OR*		
*Rhizarthrosis*		Regenerative therapy	Low level laser treatment	Fat grafting	PRP
***OR***		*OR*	*OR*	*OR*	*OR*
*Trapeziometacarpal arthritis*		Regenerative medicine	Photobio-modulation	Lipofilling	Platelet-rich plasma
***OR***			*OR*	*OR*	
*Basal thumb arthritis*			Photo therapy	Fat injection	
***OR***			*OR*		
*Arthritis of the basal thumb joint*			Light therapy		

^1^ the terms from column (1) were combined with each of the other columns (2) or (3) or (4) or (5) individually for the literature search.

**Table 2:** Study Details.

Authors	Study Type, Number of Participants	Age (Mean)	Gender	OA Stage (Eaton–Littler)	Follow-Up Period	Control Group	Technique	Injection (N, mL)
***Fat grafting***								
Erne et al., 2018	Comparative Study, *n* = 21	62.1–63.3	17 female	III–IV	18 months (mean)	Resection arthroplasty (12 patients)	Centrifugation	1.3 mL
Froschauer et al., 2021	Case series, *n* = 31	57.3–57.7	27 female	II–III	6, 24 m	-	Decanting method, mechanical homogenization	1 mL
Haas et al., 2017	Pilot study, prospective, *n* = 24	63.3	17 female	I–III	2, 6, 12 weeks	Cortisone (12)	Manual homogenization	1–1.5 mL
Haas et al., 2019	Case review, *n* = 99	61	69 female	I–III	2, 6 w 3, 6, 12 m	-	Mechanical homogenization	1–2 mL
Herold et al., 2014	Pilot study, *n*= 5	64	5 female	II–III	1, 3 m	-	Coleman technique, centrifugation	1.5 mL
Herold et al., 2017	Prospective study, *n* = 50	59.9	38 female	II–IV	6, 12 m	-	Coleman technique, centrifugation	1 mL
Meyer-Marcotty et al., 2021	Prospective study, *n* = 27	59.8	22 female	I–III	Mid-term results after 45,3 m (mean)	-	Coleman technique, centrifugation	-
***PRP + FG***								
Winter et al., 2023	RCT, *n* = 95	64	80 female	I–IV	2 w, 1, 2, 3, 24 m	PRP (24) vs. FG (25) vs. FG + PRP (25) vs. saline (21)	Fat: harvest, put away 10 min to sediment PRP: centrifugation with Endoret kit; PRP activation	FG: 1.5 mL PRP: 1.5 mL PRP + FG: 0.75 mL each
***PRP***								
Loibl et al., 2016	Pilot study, *n* = 10	56.1	8 female	II–IV	3, 6 m	-	Arthrex double-syringe system, centrifugation	1–2 mL × 2 (4 w interval)
Malahias et al., 2018	RCT, *n* = 33	62.8–63	26 female	I–III	3, 12 m	Cortisone (17)	2 consecutive, manual centrifugations; non-activated PRP	2 mL × 2 (2 w interval)
Sabah et al., 2020	Comparative study, *n* = 45	52.45	38	I–IV	4, 12 w	HA (15), cortisone (15)	Double centrifugation	1 mL × 1
Swärd et al., 2022	Retrospective study, *n* = 29 (21 CMC)	63	17 female	I–IV	3 m	-	Arthrex ACP double-syringe system, 1× centrifugation	0.5–2 mL × 2 (3–4 w interval)
***LLLT***								
Brousseau et al., 2005	RCT, *n* = 88 (3 CMC patients)	64.2–65.1	69 female	-	3, 6 w 3, 6 m	Sham LLLT (46)	GaAlAs, Eriel laser; 20 Hz modulation	3×/w for 6 w (20 min/session)
***Chondrocyte transplant***								
Messina et al., 2020	Prospective study, *n* = 10	52.4	10 female	II–III	1, 3, 6 m 1, 2, 5 y Last follow-up after 8 y (mean)	-	Cartilage fragments harvested; cells grown on collagenous biphasic matrix (MACI/Novocart)	3–4 mL of cartilage harvested; scaffold implanted once

OA: Osteoarthritis; N: Total number; RCT: Randomized controlled trial; CMC: Carpometacarpal; PRP: Platelet-rich plasma; FG: Fat grafting; LLLT: Low-level laser treatment; GaAIAs: Gallium aluminum arsenide (laser medium).

**Table 3:** Outcome.

Authors	Pain Preop (VAS or NRS)	Pain Postop (VAS or NRS)	(Q-)DASH Preop	(Q-)DASH Postop	Pinch and Grip Strength Preop (kg)	Pinch and Grip Strength Postop (kg)	Patient Satisfaction	Adverse Events (AEs)
***Fat grafting***								
Erne et al., 2018	6.4	2.9	-	24.0	-	Pinch: 0.95 (bar) Grip: 33.8 (kg) (comparable between groups)	8/10 points (satisfaction overall)	None mentioned
Froschauer et al., 2021	7.0	2.0 *	59.0	35.0 *	-	Grip 6.5 kg (n.s.)	68% of all patients satisfied	No complications observed
Haas et al., 2017	2.2 at rest, 6.8 under stress	0.2 at rest *, 3.4 under stress *	56.0	29.0 *	6.3 23	4.8 26		No adverse events observed
Haas et al., 2019	2.1 at rest, 6.6. under stress	0.8 at rest, 3.7 under stress *	-	-	5.6 25.8	5.8 25.8	73% would undergo procedure again	1 hematoma; 1 case of severe pain requiring analgesics
Herold et al., 2014	3.8 at rest, 7.4 under stress	0.8 at rest, 2.4 under stress	58.0	33.0 *	0.26 0.3	0.31 0.42	100% of patients satisfied	No complications observed
Herold et al., 2017	At rest: 3.5 (stage II), 7.6 (III), 3.7 (IV) Under stress: 7.7 (II), 7.6 (III), 8.9 (IV)	At rest: 1.0 (II), 1.8 (III), 3.0 (IV) Under stress: 2.4 (II) *, 5.6 (III) *, 6.0 (IV)	47 (stage II), 50 (III), 57 (IV)	19 (II), * 40 (III), 51 (IV)	0.3 bar (II), 0.3 (III), 0.3 (IV)	0.5 (II) *, 0.4 (III), 0.3 (IV)		2 cases of temporary paresthesia (superficial radial nerve branches) for ≤2 months
Meyer-Marcotty et al., 2021	5.9	1.9 **	50.8	29.6 **	3.7 22.2	5.1 22.8		Not mentioned
***PRP + FG***								
Winter et al., 2023	NRS at rest: 3.12 (FG) 3.12 (PRP) 3.32 (PRP + FG) NRS under stress: 6.76 (FG) 6.75 (PRP) 6.64 (PRP + FG)	NRS at rest: 1.62 (FG) 2.73 (PRP) 1.7 (PRP + FG) * NRS under stress: 3.67 (FG) 5.18 (PRP) 3.09 (PRP + FG) *	-	Median reduction of Q-DASH: −14.8 (FG) −9.1 (PRP) −15.9 (PRP + FG)	Pinch: 1.28 FG 1.58 PRP 1.46 PRP + FG Grip: 20.76 FG 22.87 PRP 20.78 PRP + FG	Pinch: 1.41 FG 1.78 PRP 1.71 PRP + FG Grip: 20.55 FG 21.32 PRP 22.17 PRP + FG		1 hematoma, no serious AEs associated with intervention
***PRP***								
Loibl et al., 2016	6.2	5.4 *	32.9	26.8	6.0 16.4	4.9 16.7	No patient dissatisfied	1 palmar wrist ganglion ^2^
Malahias et al., 2018	7.5	2.0*	50.4	20.4 *	-	-	69 % satisfied *	Not mentioned
Sabah et al., 2020	8	4 after 4 w 5 after 12 w	-	-	-	Improved after 4 w *		Not mentioned
Swärd et al., 2022	2 at rest 8 under stress (NRS)	1 6	-	54	5.5 23	6.0 23	-	No AEs
***LLLT***								
Brousseau et al., 2005	2.36 (AUSCAN)	1.94 (AUSCAN) VAS n.s. ^1^	-	-	-	Grip strength improved *^, 1^	-	1 erythema 1 other ^3^
***Chondrocyte transplant***								
Messina et al., 2020	8	1 *	55	7.3 *	Pinch 3.76 Grip: 22	6.25 * 28.5	-	No complications

* *Statistically significant results*; ** *highly significant*; (Q-)DASH: (Quick) Disabilities of the Arm, Shoulder and Hand Questionnaire; VAS: Visual analogue scale; NRS: Numeric rating scale; AUSCAN: Australian Canadian Osteoarthritis Index; ^1^ data not shown; n.s.: non-significant; ^2^ resolved without intervention; ^3^ reoccurrence of menstruation in 1 perimenopausal patient.

## Data Availability

The data that support the findings of this study were obtained from online databases (PubMed, OVID), journal websites or other research platforms where restrictions or charges may apply. Such datasets may be requested from the respective journals or by contacting the authors directly.
